# Zero-Valent Iron and Sand Filtration Reduces Levels of *Cyclospora cayetanensis* Surrogates, *Eimeria tenella* and *Eimeria acervulina*, in Water

**DOI:** 10.3390/microorganisms12112344

**Published:** 2024-11-16

**Authors:** Alan Gutierrez, Matthew S. Tucker, Christina Yeager, Valsin Fournet, Mark C. Jenkins, Jitender P. Dubey, Kalmia E. Kniel, Benjamin M. Rosenthal, Manan Sharma

**Affiliations:** 1 Environmental Microbial and Food Safety Laboratory, Beltsville Agricultural Research Center, Northeast Area, Agricultural Research Service, U.S. Department of Agriculture, Beltsville, MD 20705, USA; alan.gutierrez@usda.gov; 2 Animal Parasitic Diseases Laboratory, Beltsville Agricultural Research Center, Northeast Area, Agricultural Research Service, U.S. Department of Agriculture, Beltsville, MD 20770, USA; matthew.tucker2@usda.gov (M.S.T.); christina.yeager@usda.gov (C.Y.); valsin.fournet@usda.gov (V.F.); mark.jenkins@usda.gov (M.C.J.); jitender.dubey@usda.gov (J.P.D.); benjamin.rosenthal@usda.gov (B.M.R.); 3 Department of Animal and Food Sciences, University of Delaware, Newark, DE 19716, USA; kniel@udel.edu

**Keywords:** *Cyclospora cayetanensis*, zero-valent iron, *Eimeria*, water treatment, irrigation water, produce safety

## Abstract

Recurring outbreaks of cyclosporiasis linked to fresh produce demonstrate the need to develop interventions to reduce *C. cayetanensis* in irrigation water. *C. cayetanensis* is resistant to commonly used irrigation water treatments, such as chemical sanitizers, making removal of oocysts by filtration the most suitable intervention. This study evaluated the reduction of *Eimeria tenella* and *E. acervulina*, as surrogates for *C. cayetanensis*, in water using filters packed with sand alone or mixtures of sand and zero-valent iron (ZVI). Water inoculated with *Eimeria* spp. oocysts was filtered through laboratory-scale (PVC column) and field-scale (swimming pool filter) filters packed with either 100% sand or 50% ZVI/50% sand (*v*/*v*). Filtered and backflush water was examined microscopically for oocysts. Laboratory-scale filters with 50% ZVI significantly (*p* < 0.05) reduced 99.9% of *E. tenella* oocysts compared to 55.3% with filters containing 100% sand. At the field-scale level, 50% ZVI filters significantly (*p* < 0.05) reduced 70.5% of *E. acervulina* oocysts compared to 54.5% by 100% sand filters. Filters were backflushed to examine the recovery of these parasites during routine filter-media cleaning procedures. Backflush recovery of oocysts ranged from 4.42–16.7%. The addition of ZVI significantly improved the reduction of *Eimeria* spp. oocysts at both filter scales. and should be further investigated as a potential irrigation water intervention to reduce *C. cayetanensis*.

## 1. Introduction

*Cyclospora cayetanensis*, a human coccidian intestinal parasite, is a recurring cause of human diarrheal illnesses worldwide. While numerous species of *Cyclospora* have been reported in animals, *C. cayetanensis* is the only species known to infect humans. *C. cayetanensis* infections are commonly caused by the consumption of food or water contaminated with infective (i.e., sporulated) oocysts [1]. Symptoms typically begin within 1 week of ingestion, with onset ranging from 2 days to 2 weeks, and include diarrhea, abdominal cramping, nausea, flatulence, fatigue, loss of appetite, weight loss, and low-grade fever [2]. Outbreaks of cyclosporiasis are endemic to Central and South America, Southeast Asia, Egypt, Turkey, and the Indian subcontinent [1,3]. Cyclosporiasis demonstrates varied seasonality across endemic regions, where optimal environmental factors (e.g., rainfall, temperature, and humidity) may coincide with human activities that lead to parasite exposure and outbreaks [1]. In the US, cases of cyclosporiasis generally occur between March and September, with infections peaking in July [4].

Since the mid-1990s, *C. cayetanensis* has caused numerous foodborne outbreaks in the US and Canada associated with fresh produce [4]. Basil, cilantro, leafy greens, raspberries, and blackberries have each been linked to several outbreaks [5]. In 2020, a multistate outbreak of cyclosporiasis, linked to bagged salad products caused 701 illnesses and 38 hospitalizations across 14 states in the US [6]. Between 2018 and 2023, over 1000 laboratory-confirmed cyclosporiasis cases were reported each year in the US [4,7,8]. Historically, these US outbreaks have been associated with produce imported from Mexico, Guatemala, and Peru [1,5]. However, in 2018, a microbiological survey of fresh herbs conducted by the US Food and Drug Administration (FDA) reported the detection of *C. cayetanensis* in a sample of US-grown cilantro. A subsequent sample collected from the implicated farm was also positive for *C. cayetanensis* [9]. During their investigation of the 2020 multistate outbreak in bagged salads, FDA detected *C. cayetanensis* in canal water samples collected near implicated farms in Florida. However, they were unable to determine if the *C. cayetanensis* detected was a genetic match to the outbreak cases [6]. A 2022 meta-analysis of *C. cayetanensis* prevalence in water estimated a prevalence of 3.46% in North America based on water samples collected in six studies [10]. Recent evidence from surveys and outbreak investigations may suggest the existence of an emerging domestic reservoir for *C. cayetanensis* in the US.

The pre-harvest contamination of fresh produce likely occurs through contact with soil or water containing sporulated oocysts [1]. While good agricultural practices, water sanitation, and worker health and hygiene practices may reduce the risks of parasite transmission and exposure, they cannot eliminate the risks of outbreaks entirely [5]. Several interventions have been studied for their potential to control *C. cayetanensis* in food or water. Studies exposing oocysts to various pesticides (30 min to 24 h) or gaseous chlorine dioxide (4.1 mg/L for 20 min) reported no decreases in sporulation [11,12]. However, microwave heating (up to 45 s), exposure to magnesium oxide (MgO) nanoparticles (15 mg/mL for 24 h; 12.5 mg/mL for 72 h), and treatment with sodium dichloroisocyanurate (NaDCC) (1 g/L for 1–2 h) have demonstrated significant reductions in oocyst sporulation [13,14,15]. Sathyanarayanan and Ortega [16] reported that sporulation of *C. cayetanensis* in water and on basil could be stopped by exposure to −70 °C for 60 min or 70 °C for 15 min. Given that *C. cayetanensis* oocysts are highly resistant to chemical disinfectants commonly used in the food industry [17], there is an urgent need for practical, effective interventions that can reduce the risk of pre-harvest contamination.

Slow sand filtration is a simple, low-cost technique to improve drinking and agricultural water quality by reducing levels of bacteria, viruses, and parasites [18,19,20]. In modern agriculture, rapid sand filters are commonly used in drip irrigation systems to prevent drip lines and emitters from clogging by reducing turbidity and organic matter from irrigation water sources (e.g., ponds and rivers) [21]. Studies incorporating zero-valent iron (ZVI), a low-cost reactive metal, into sand filters have demonstrated its ability to enhance reductions of nitrates, antimicrobial compounds, bacteria, viruses, and parasites in water [22,23,24,25,26,27,28,29,30,31,32,33,34]. Kniel [22] and Yeager et al. [26] reported significantly greater reductions (>99%) of *Cryptosporidium*, *Eimeria* spp., and *C. cayetanensis* in water-filtered ZVI/sand filters compared to sand alone. While much of this work has been performed using small, laboratory-scale filters, it nonetheless suggests that ZVI and sand filters may be an effective, low-cost intervention for improving irrigation water quality in the field.

The inability to propagate *C. cayetanensis* in animal models or laboratory culture constitutes a major barrier for conducting such studies at scale [35]. Oocysts can only be obtained from clinical stool samples collected from infected humans [4]. This challenge highlights the need for surrogate organisms that can be used to study *C. cayetanensis*. Coccidian parasites in the genus *Eimeria* infect numerous host species, such as cattle, poultry, mice, and rabbits [36]. However, they do not infect humans. Phylogenetic analyses have demonstrated that *Cyclospora cayetanensis* is closely related to several *Eimeria* spp. that infect poultry [37,38]. Their genetic relatedness, non-infectivity with regard to humans, and the ability to produce high levels of oocysts in chickens, makes them useful surrogates for *C. cayetanensis* [39]. Several studies have used *E. acervulina* and *E. tenella* as surrogates for *C. cayetanensis* on foods or in water to examine different interventions. These include gamma irradiation of raspberries [40], UV radiation and high hydrostatic-pressure processing of basil and raspberries [41], ozone treatment of water [42], and ZVI/sand filtration of water [22,26].

Evidence on the efficacy of parasite removal by ZVI/sand filtration may be limited, but current findings suggest that it may be a promising intervention for improving water quality and reducing the risk of foodborne parasites that contaminate fresh produce. However, the efficacy of ZVI in rapid sand filters, common in agricultural production, to reduce foodborne parasites in water has not been previously demonstrated. Thus, the objectives of this study were to evaluate the reduction of *Eimeria* spp. oocysts in water using laboratory and field-scale filtration systems filled with sand alone, or with ZVI/sand mixtures, and to examine the recovery of these parasites in water after backflushing the filters. Although oocysts of *E. tenella* (22 × 19 µm) and *E. acervulina* (18 × 15 µm) [43] are slightly larger than those of *C. cayetanensis* (8–10 µm) [1], they nonetheless serve as useful surrogates for evaluating filter efficacy [22,26]. *E. tenella* was used to test laboratory-scale filters and *E. acervulina* was used to test field-scale filters packed with 100% sand or 50% ZVI/50% sand.

## 2. Materials and Methods

### 2.1. Experimental Design

**Figure 1:**
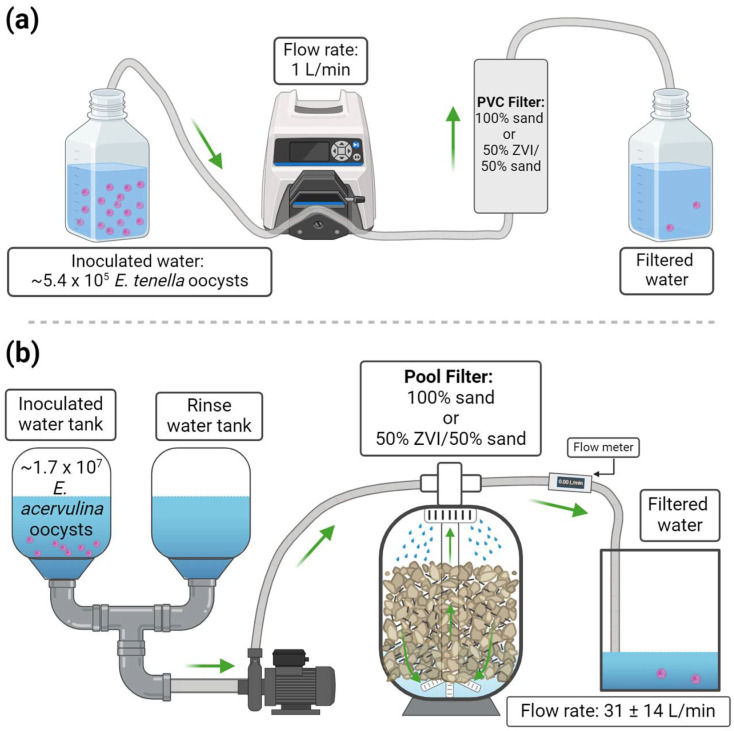
Overview of filtration experiments. (**a**) Lab-scale PVC filter (n = 3) and (**b**) field-scale pool filter packed with 100% sand or 50% ZVI/50% sand (n = 3). Green arrows indicate the direction of water flow during filtration. Created with BioRender.com.

The efficacy of two ZVI/sand filter types was tested as shown in Figure 1. The two filter types were a laboratory-scale, polyvinyl chloride (PVC) column (2″ diameter × 4.25″ length) and a field-scale, consumer-grade swimming pool sand filter (10″ diameter × 10.25″ length). Agricultural sand filters and swimming-pool sand filters are both examples of pressurized rapid sand filters that push water down through a bed of filter media to remove organic and inorganic particles from water [44,45]. Given their similar filtration mechanics, a swimming pool filter was used to simulate a field-scale agricultural filtration system. Both filter designs followed similar experimental procedures: (1) filtration of uninoculated water, (2) filtration of inoculated water, followed by (3) another uninoculated water flush, and (4) backflushing filters with uninoculated water. In each experiment, filtered and backflush water was collected to enumerate *Eimeria* spp. oocysts.

### 2.2. Construction of Sand and ZVI Filters

#### 2.2.1. Laboratory-Scale Filter

**Figure 2:**
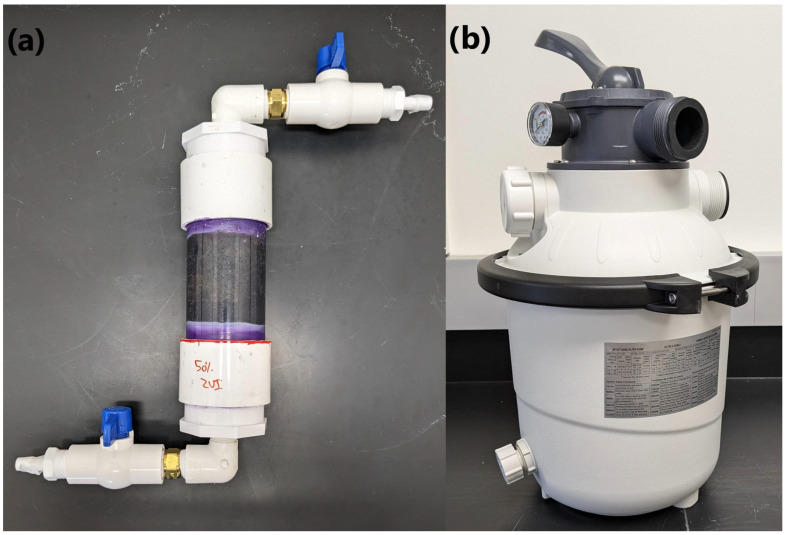
Constructed zero-valent iron and sand filters. (**a**) Lab-scale PVC filter (2″ diameter × 4.25″ length); (**b**) field-scale swimming pool filter (10″ diameter × 10.25″ height).

Laboratory-scale filters, packed with either 100% sand or 50% ZVI/50% sand (*v*/*v*), were constructed using PVC pipes and fittings. A 2″-diameter × 4.25″-length PVC plain-end pipe was capped on both ends by priming and gluing a 2-inch PVC snap-in drain (model# 435612; Oatey, Cleveland, OH, USA) onto each end. Superglue was used to attach a 2.76″-diameter circle of landscaping fabric onto the drain fitting on one end of the pipe. A 2″ × 2″ PVC coupling was primed and glued onto the end of the pipe with the landscaping cloth attached, followed by attaching a 2″ × ½″ reducing bushing (male spigot x female NPT) onto the PVC coupling. The completed filter end was capped using a ½″ threaded PVC plug and allowed to dry for 24 h before packing with sand and/or ZVI. Filters were wet-packed using sterile deionized water and the filtration media was added through the open drain fitting. The filter media, comprised of sand (particle size: 0.45–0.55 mm; Northern Filter Media, Muscatine, IA, USA) alone or a mixture of 50% sand and 50% ZVI (*v*/*v*) (particle size: 0.43–0.60 mm; Winoa USA, Melvindale, MI, USA) was used to fill the entire filter body. Sand and ZVI were pre-mixed before being added to the filter. The open end of the filter was sealed by gluing on a 2.76″-diameter circle of landscaping fabric onto the open drain fitting and attaching a PVC coupling and reducing bushing as previously described. Both ends of the filter were capped with a ½″ threaded PVC plug until its use. These PVC column filters had a filter bed volume (i.e., the volume of media in the filter) of 219 mL, with a pore volume (i.e., the volume of void space in the media) of 77 mL for 100% sand filters (35% porosity) and 101 mL for 50% ZVI/50% sand (46% porosity) filters. An example of a constructed lab-scale PVC filter is provided in Figure 2a.

#### 2.2.2. Field-Scale Filter

Field-scale filters were constructed using a consumer-grade sand pool filter (Krystal Clear Sand Filter Pump; item no. 26643EG; Intex Recreation Corp., Long Beach, CA, USA), with a 10″-diameter × 10.25″-height filter tank, according to the manufacturer’s instructions. The filter tank was filled with sand (particle size: 0.45–0.55 mm) alone or a mixture of 50% sand and 50% ZVI (*v*/*v*) (particle size: 0.075–1.18 mm; CC-1190; Connelly-GPM, Inc., Chicago, IL, USA). Sand and ZVI were pre-mixed before being added to the filter. Filters were wet-packed using sterile deionized water. These pool filters had a filter bed volume of 8.20 L, with a pore volume of 2.87 L for 100% sand filters (35% porosity) and 3.28 L for 50% ZVI/50% sand filters (40% porosity). An example of a constructed field-scale pool filter is provided in Figure 2b.

### 2.3. Propagation and Preparation of Eimeria spp. Inoculum

Filtration experiments used *E. tenella* and *E. acervulina* as surrogate organisms for *C. cayetanensis*. *Eimeria* oocysts were obtained from propagations performed according to Ryley et al. [46]. Briefly, oocysts were separated from the feces of experimentally infected chickens via saturated-salt (NaCl) floatation and centrifugation. Recovered oocysts were sporulated at 29 °C for 1–3 days in 2% (*v*/*v*) potassium dichromate, and then stored at 4 °C. In preparation for experimental use, oocysts were separated from potassium dichromate via centrifugation (3309× *g*, 5 min) and washed with deionized water several times. Washed oocysts were resuspended in 2 mL of 6% sodium hypochlorite (bleach) solution and incubated on a rocking mixer (24 rpm) for 15 min at room temperature. Treatment with sodium hypochlorite was performed to reduce bacterial and fungal contamination of oocysts. Oocysts were centrifuged (3309× *g* for 5 min) and washed twice with deionized water, before being resuspended in 50 mL of deionized water. This final oocyst suspension was stored at 4 °C until use. Oocyst concentrations (oocysts/mL) were determined using a hemocytometer. Laboratory (lab)-scale filter experiments were performed with bleached and sporulated *E. tenella* oocysts, whereas field-scale filter experiments were performed with bleached and sporulated *E. acervulina* oocysts.

### 2.4. Animal Ethics Statement

This study was conducted following the animal use protocol (22-06) approved by the BARC Institutional Animal Use and Care Committee, United States Department of Agriculture. The chickens utilized in this study exhibited no outward signs of severe disease throughout the study. Chickens were humanely euthanized after the study’s conclusion and all efforts were made to minimize animal suffering.

### 2.5. Water Inoculation and Filtration

#### 2.5.1. Lab-Scale Filter Experiments

The PVC filters were prepared for each experiment (Figure 1a) by removing the ½″ PVC plugs from each end and attaching the following fittings, in order, on both ends: ½″ × ½″ 90° street elbow (FNPT × MNPT), ½″ × ½″ hex nipple, ½″ × ½″ 2-way ball valve (FNPT × FNPT), and a ½″ barbed-hose adapter for 3/8″ inner diameter (ID) tubing. Polytetrafluoroethylene (PTFE) tape was used on all threaded fittings to prevent leaks. Filters were vertically attached to a stand and fitted with 9.7 mm ID platinum-cured silicone tubing (Masterflex L/S 36, Avantor, Radnor, PA, USA) on both ends of the filter. All silicone tubing was autoclaved before each filtration trial.

Internal tubing of the peristaltic pump was rinsed with 500 mL of 70% ethanol and 2 L of sterile deionized water at the start of each trial. Tubing at the bottom of the filter was connected to a peristaltic pump (model: 74203-03; Cole-Parmer, Vernon Hills, IL, USA) which pumped water from the bottom up through the filter for each trial. The peristaltic pump was set to pump at 1 L/min. Tubing at the top of the filter was used to collect filtered water. Filters were pre-rinsed with 1 L of sterile deionized water, then inoculated with 5.4 × 10^5^ *E. tenella* oocysts suspended in 100 mL of deionized water and rinsed with 1 L of sterile deionized water. Filtered water was collected in six separate fractions (one 100 mL fraction and five 200 mL fractions), beginning when the inoculated water entered the filter. After the ca. 2.1 L volume had been filtered, the filter was then backflushed with 1 L of sterile deionized water, collected in a sterile 1 L bottle. Between each replicate filter, the peristaltic pump’s internal tubing was rinsed with 1 L of sterile deionized water. Sterile silicone tubing connecting to and from the filter was also changed for each replicate filter. Filtration and backflush experiments were performed in triplicate (n = 3) for both 100% sand and 50% ZVI/sand filters using three separate filters of each type.

#### 2.5.2. Field-Scale Filter Experiments

The 100%-sand and 50%-ZVI/sand pool filter experiments used two, 15-gallon (57 L) cone bottom tanks (part no. 016211; Tamco Industries, Lima, OH, USA) for water inputs (Figure 1b). Each tank was fitted with 2″ PVC elbows and connected with 1.5″ polyethylene (PE) hoses (item no. 11009; Intex Recreation Corp.) to 2-way 2″ PVC valves affixed to a 2″ PVC tee. These valves were used to switch between water inputs from both tanks. The PVC tee was connected by a PE hose to a centrifugal water pump (item no. 12703; Intex Recreation Corp.). The water-outlet hose for the pool filter was connected to a 1.5″ digital water-flow meter (model: TM15SQ9GMB; Great Plains Industries, Inc., Wichita, KS, USA). The flow meter was connected by a hose to another 2″ PVC tee with 2-way valves on both ends and hoses to direct output water. These valves were used to switch between water collection and wastewater sent to a drain. In each filtration trial, one water tank was used to hold water for the initial backflush and rinse of the pool filter, and the other tank was used for the inoculated water and post-inoculum rinse.

Filtration trials began with a 30 L backflush and 15 L rinse with municipal water to precondition the filter. This was followed by the filtration of 10 L of municipal water containing 1.7 × 10^7^ *E. acervulina* oocysts and 15 L of uninoculated municipal water. The filter was then backflushed with 10 L of uninoculated municipal water. Filtered water (10 L inoculum and 15 L rinse) and backflush water (10 L) were collected in separate 10-gallon (37.9 L) tanks (part no. 004955; Tamco Industries). Water-collection tanks were mixed before taking three 250 mL samples from each for oocyst enumeration. In these experiments, three separate pool filters of each media type (100% sand and 50% ZVI/sand) were constructed and tested with a single filtration and backflush event (n = 3). The flow meter did not have a data logging function, and thus video of the flow meter’s screen was recorded to collect flow rate data. Videos of filtration trials were analyzed, and flow rates were recorded every five seconds.

### 2.6. Eimeria Oocyst Enumeration

Filtration and backflush water samples were concentrated by gravity settling for at least 12 h at 4 °C. After settling, the supernatant was carefully removed without disturbing the settled oocysts. This settling process was repeated until samples were sufficiently concentrated to count the oocysts. Fraction samples from lab-scale sand filters were concentrated to 35–45 mL for filtered water and 130 mL for backflush water. Fractions collected from lab-scale 50%ZVI filters were concentrated to 300 µL for filtered water and 27–39 mL for backflush water. Pool filter samples were concentrated to 100 mL.

Concentrated samples were mixed with 2M sucrose at a 1:1 ratio and enumerated by microscopy using a McMasters chamber at 100× total magnification. Oocyst concentration (oocysts/mL) was calculated using the following formula: (*N_o_* × 2)/0.15, where *N_o_* is the total number of oocysts counted in 6 lanes of the McMasters chamber. The total number of oocysts in each sample was then calculated as follows: oocysts/mL × *V_T_*, where *V_T_* is the total volume collected of either filtered or backflush water.

### 2.7. Statistical Analysis

Oocyst reduction and recovery are stated as percentages of oocysts compared to the initial inoculum concentration. Welch’s two-sample *t*-test was used to compare the filtration reduction and backflush recovery of *Eimeria* oocysts between filter types (100%-sand and 50%-ZVI/50% sand) in the lab- and field-scale experiments. Pairwise Welch’s two-sample *t*-tests were also used to compare oocyst recovery after filtration for each of the six fractions collected during the lab-scale experiments and the Benjamini–Hochberg procedure [47] was used to adjust *p*-values for multiple comparisons. Statistical analyses and visualizations were performed in R version 4.4.1 (http://www.r-project.org/, accessed on 27 October 2024) with the significance level set at α = 0.05.

## 3. Results

### 3.1. Lab-Scale Filters

**Figure 3:**
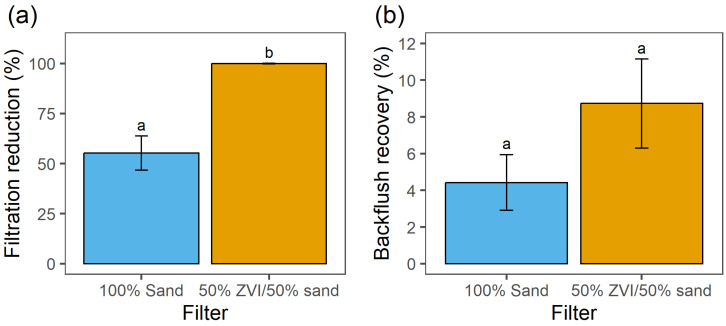
(**a**) Reduction (%) of *E. tenella* oocysts in filtered water and (**b**) recovery (%) of *E. tenella* oocysts in backflush water from 100%-sand (blue) and 50%-ZVI/50% sand (orange) lab-scale PVC filters. Bars represent means ± standard deviations (n = 3). Bars with the same letter are not significantly different (*p* > 0.05).

The reduction of *E. tenella* oocysts was significantly greater (*p* < 0.05) after filtration through 50%-ZVI filters compared to 100%-sand filters. Oocysts were reduced by 99.9% after 50%-ZVI filtration, whereas 100%-sand filtration reduced oocysts by 55.3% (Figure 3a). Oocysts were recovered in all six fractions of filtered water from each filter type, but fractions 2 and 3 contained the most oocysts (Table 1). The 100%-sand filters yielded 28.84% and 11.94% recovery in fractions 2 and 3, respectively. However, from 50%-ZVI filters, fractions 2 and 3 yielded only 0.06% and 0.03% recovery, respectively. Pooling oocysts from fractions 2–5, significantly more oocysts were recovered from 100% sand than from 50% ZVI (*p* < 0.05). The percent of oocysts recovered from the backflush did not differ significantly for 50%-ZVI filters (8.73%) and 100%-sand filters (4.42%) (*p* > 0.05) (Figure 3b).

### 3.2. Field-Scale Filters

**Figure 4:**
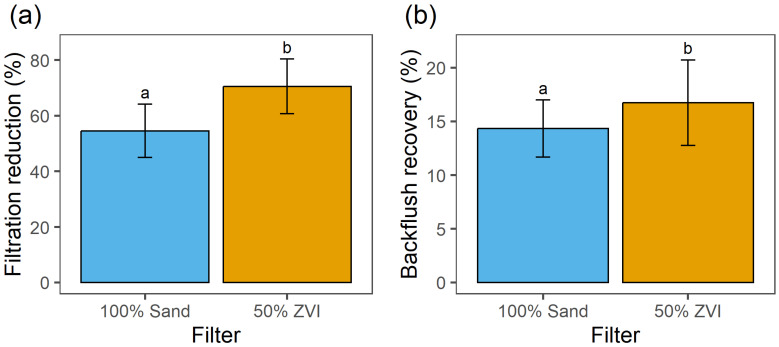
(**a**) Reduction (%) of *E. acervulina* oocysts in filtered water and (**b**) recovery (%) of *E. acervulina* oocysts in backflush water from 100%-sand (blue) and 50%-ZVI/50% sand (orange) field-scale pool filters. Bars represent means ± standard deviations (n = 3). Bars with the same letter are not significantly different (*p* > 0.05).

The reduction of *E. acervulina* oocysts was significantly greater (*p* < 0.05) after filtration through 50%-ZVI compared to 100%-sand pool filters (Figure 4a). The mean oocyst reduction was 70.5% in 50%-ZVI filters and 54.5% in the 100%-sand filters. Backflush recovery was significantly higher (*p* < 0.05) in 50%-ZVI filters (16.7%) compared to the 100%-sand filters (14.3%) (Figure 4b). The mean flow rate for the 100%-sand and 50%-ZVI pool-filter trials was 31 ± 14 L/min (Figure 1b).

## 4. Discussion

Several studies have demonstrated the efficacy of ZVI/sand filtration in improving microbial water quality through the removal of bacteria and viruses [23,24,27,28,29,31,32,33,34,48]. However, studies on ZVI/sand filtration of parasites have been limited [22,26]. This study sought to evaluate the efficacy of laboratory and field scale filters, using sand alone or sand with 50% ZVI (*v*/*v*), to remove *Eimeria* oocysts from water. Additionally, filters were backflushed to examine the recovery of oocysts as a result of cleaning the filter media.

The addition of ZVI to both laboratory- and field-scale filters significantly improved their ability to remove oocysts from water. The greatest reductions achieved in this study were a 99.9% reduction of *E. tenella* in 50%-ZVI lab-scale filters and a 70.5% reduction of *E. acervulina* in 50%-ZVI field-scale filters. Kniel [22] reported reductions of 99.3% and 99.9999% for *E. tenella* oocysts in sand and 50%-ZVI filters, respectively (113 mL filter media; 1 mL/min). Yeager et al. [26] also achieved ≥ 99% reductions of *E. tenella* and *E. maxima* in 50%-ZVI mini filters (24.5 mL filter media) and *E. acervulina* in 35%-ZVI pool filters (8.2 L filter media; 9.7 L/min). Most notably, Yeager et al. [26] also reported a 97% removal of *C. cayetanensis* oocysts in 35%-ZVI mini filters (24.5 mL filter media), demonstrating comparable behavior to *Eimeria* oocysts and further supporting the use of *Eimeria* spp. as surrogates for filtration studies. The higher reductions (>99%) reported by Kniel [22] for both sand and ZVI/sand filters compared to this study, and Yeager et al. [26], are likely due to the slow flow rate (1 mL/min) used in their study. This slow flow rate may have allowed these filters to behave more similarly to slow sand filters, where a combination of physical (size exclusion/sedimentation), chemical (adsorption/attachment), and biological (predation/degradation) mechanisms are involved in removal during filtration [19,20]. The extended contact time allowed by the slow flow rate likely led to greater attachment of oocysts to the filter media. Given the higher flow rates used in this study (1 and 31 L/min), the dominant mechanisms for parasite removal were likely physical size exclusion and chemical adsorption of oocysts to sand and ZVI particles. Yeager et al. [26] observed iron particles attached to the surfaces of filtered *Eimeria* oocysts, suggesting that the increased removal of oocysts is due to their adsorption to ZVI particles in the filter.

Flow rate was determinative in explaining the efficacy of ZVI/sand filters to bind and remove parasite oocysts from water. Using gravity filtration, Yeager et al. [26] achieved >99% reduction of *Eimeria* oocysts using a 35%-ZVI pool filter (8.2 L filter media), similar to the pool filter used in this study, and recorded a mean flow rate of 9.7 L/min. In this study, operating the 50%-ZVI pool filters at a higher flow rate (31 L/min) lessened the reduction, to 70.5%. Similarly, a study on the removal of *Cryptosporidium*-sized microspheres (4.5 µm diameter) from swimming pools via sand filtration reported a decrease in microsphere removal from >90% to 50% when filtration rates were increased from 30 m/h to 37 m/h [49]. Using a slower flow rate for the 50%-ZVI pool filters in this study would likely have resulted in greater reductions of oocysts by allowing more residence time for the water, facilitating greater attachment of oocysts to the filter media. Adapting ZVI/sand filtration for use in commercial agriculture will necessitate increasing flow rates and filter sizes to meet the water demands of large fields. However, more design improvements and investigations are required to appropriately scale up filter size and flow rate to maintain high parasite reductions (≥99%). The relationship between filter size, flow rate, and parasite reduction efficacy therefore requires further investigation.

Water filtered through the lab-scale filters was collected in six separate fractions to examine the dynamics of oocyst passage through each filter. The first collected fraction (100 mL) represents the collection of uninoculated water left in the filter after the initial water flush. The recovery of oocysts in the first fraction of the 50%-ZVI filter (101 mL pore volume) indicates that the displacement of water during filtration does not occur uniformly, and that mixing and preferential flow may have occurred between water stored in the filter and the inoculated water. In both sand and 50%-ZVI lab-scale filters, the highest level of oocysts occurred in fraction 2, which represents a mixture of the inoculum water and the first pore volume of flush water. However, fraction 2 recovery in the 50%-ZVI filter (0.06%) was significantly lower than the 100%-sand filter (28.84%). Yeager et al. [26] observed an identical trend in the recovery of oocysts through their mini filters, where the fraction containing the first pore volume of flush water after inoculation of the filter contained the highest level of oocysts. In both studies, oocyst counts in filtered fractions decreased gradually after the first pore volume flush (fraction 2 in this study). Oocyst recovery in fractions 3–5 for 50% ZVI were also significantly lower compared to 100% sand, demonstrating that filters containing ZVI have enhanced binding capacity compared to sand filters.

Filter media (sand, or sand and ZVI) is cleaned by backflushing, or backwashing, which involves reversing the flow of water through the filter media bed to dislodge and remove captured debris [50]. This practice prevents sand filters from becoming clogged over time and allows the media to be used for longer durations before being replaced. Oocyst recovery from filter backflushes ranged from 4.42% to 16.7% for all filters tested in this study. Backflush recovery was highest in 50%-ZVI filters at both lab (8.73%) and field scales (16.7%). The higher recovery of oocysts from the 50%-ZVI filter backflush may simply be due to the higher retention of oocysts during filtration, which are then released during backflushing. Studies of swimming-pool sand filters and rapid sand filters used for drinking water production have detected *Giardia* and *Cryptosporidium* in backflush waters [51,52]. Kniel [22] reported that *C. parvum* oocysts remained infectious in cell culture assays after slow filtration through 50%-ZVI and sand filters. Therefore, the release of oocysts during routine backflushing of filters risks discharging viable parasites back into the environment. Filter operators should carefully consider how and where to discharge backflush water to minimize the potential risks posed by this essential maintenance process. Overall, it was expected that some oocysts would be recovered from the backflush, and results here indicate ZVI/sand filtration captured more oocysts during filtration than those released during backflushing.

Commercial adoption of ZVI/sand filtration by growers will require further research in four key areas: flow rate, influent water quality, ZVI particle size, and filter longevity. As previously discussed, flow rate is key in determining the removal efficacy of a filter. Previous studies on the removal of bacteria, viruses, and parasites using ZVI/sand filters have used flow rates ranging from 1 mL/min to 9.7 L/min [22,23,24,25,26,28,29,31,32,33,34]. This study represents the highest flow rate (31 L/min) tested using a ZVI/sand filter. The higher oocyst removal achieved by Yeager et al. [26] using the same pool filter design with 35% ZVI suggests that an optimal flow rate for the 50%-ZVI pool filter used in this study may be somewhere between 9.7 and 31 L/min. Future studies should seek to determine an optimal filter-size-to-flow-rate ratio for ZVI/sand filters that can be used to develop larger filters that can meet agricultural industry needs.

The influence of different water quality parameters (e.g., pH, turbidity, dissolved oxygen, conductivity) on ZVI/sand filtration efficacy will need to be evaluated. Given that other water treatments, such as chemical sanitizers or ultraviolet light, are not suitable for all water types [53], the performance of ZVI/sand filtration may vary, based on the type of water being filtered. Yeager et al. [26] reported that simulated agricultural water (containing humic acid and sea salts) decreased the performance of sand mini filters, but increased the performance of 50%-ZVI mini filters. Kim et al. [32] found that higher turbidity and conductivity in influent water led to lower *Escherichia coli* reductions in their ZVI/sand filters. Previous studies of *Cryptosporidium* have demonstrated that filter retention is influenced by pH, the ionic strength of the solution, and dissolved organic compounds (e.g., humic and fulvic acids), which affect the surface properties of oocysts and their ability to attach to filter media particles [54,55,56,57,58,59]. Turbidity in water can also help improve filtration efficacy by reducing the size of pores in the filter bed, as larger particles are trapped over time, improving the retention of smaller particles [50,60]. However, too much turbidity will undoubtedly clog the filter faster and require more frequent backflushing. Additionally, effluent water quality should also be considered. Previous studies have reported that the levels of potential ZVI-filtration byproducts, such as residual iron and heavy metals (e.g., chromium, lead, cadmium, and arsenic) in ZVI-filtered water were below US drinking water standards [24,61].

Most of the previous studies on ZVI/sand filtration have used ZVI with particle sizes (grain sizes) of either 0.43–0.80 mm [27,28,29,30,31,32] or 0.14–4.76 mm [22,23,24,33,62]. This study used two different ZVI particle size ranges, 0.43–0.60 mm for the lab scale and 0.075–1.18 mm for the pool filters. Studies filtering *Cryptosporidium* found that decreasing the grain size of sand filter media increased oocyst removal [56,63]. Decreasing the grain sizes of sand and ZVI in filters would diminish pore sizes, likely enhancing oocyst retention. However, the downstream effects that this would have on achievable flow rates and filter-media longevity remain unclear. Marik et al. [29] observed consistent removal of *E. coli* (2.27 ± 0.27 log CFU/mL) and *Listeria monocytogenes* (2.53 ± 0.31 log CFU/mL) after filtration of 390 L of water using 35% ZVI/65% sand filters (1.24 L filter media) over a 22-week period. The longevity of ZVI/sand filter media and its ability to consistently remove parasite oocysts over hundreds or thousands of liters of water has not been studied.

## 5. Conclusions

Water filtration through ZVI/sand filters significantly enhanced the removal of coccidian parasites *E. tenella* and *E. acervulina*, as surrogates for *C. cayetanensis*. Swimming pool filters, which function similarly to agricultural rapid sand filters, provide a scalable filtration model useful for optimizing the performance of ZVI/sand filters. Flow rate during filtration influenced the extent of parasite removal, where increased flow rates resulted in lower reductions in filtered water. Backflushing filters dislodged some of the captured oocysts from the filter media. Future studies should address issues surrounding usable flow rates, the effects of influent water quality, optimal ZVI particle size, and filter longevity to help growers assess the suitability of ZVI/sand filtration for their operations. The findings of this study provide strong evidence that ZVI/sand filtration can be a useful intervention for irrigation water to reduce the risk of *C. cayetanensis* contamination in the pre-harvest growing environment.

## Figures and Tables

**Table 1:** Recovery (%) of *E. tenella* oocysts in each fraction of filtered water collected during lab-scale PVC filter experiments.

Fraction	Volume (mL)	100% Sand	50% ZVI/50% Sand
1 ^a^	100	0.05 ± 0.03 a	0.004 ± 0.006 a
2	200	28.84 ± 5.16 a	0.06 ± 0.08 b
3	200	11.94 ± 3.14 a	0.03 ± 0.03 b
4	200	2.38 ± 0.23 a	0.01 ± 0.02 b
5	200	0.91 ± 0.20 a	0.003 ± 0.003 b
6	200	0.60 ± 0.27 a	0.01 ± 0.02 a

^a^ The first fraction was collected as soon as the inoculated water entered the filter and was intended to collect the uninoculated water in the filter as it was displaced by the inoculated water. Values represent percent recovery (%) mean ± standard deviation (n = 3). Means with the same letter in each row are not significantly different (*p* > 0.05).

## Data Availability

The original contributions presented in the study are included in the article, further inquiries can be directed to the corresponding author.
